# Transcription Factors in Cancer Development and Therapy

**DOI:** 10.3390/cancers12082296

**Published:** 2020-08-15

**Authors:** Kanchan Vishnoi, Navin Viswakarma, Ajay Rana, Basabi Rana

**Affiliations:** 1 Department of Surgery, Division of Surgical Oncology, University of Illinois at Chicago, Chicago, IL 60612, USA; kvishnoi@uic.edu (K.V.); navinv@uic.edu (N.V.); arana@uic.edu (A.R.); 2 University of Illinois Hospital and Health Sciences System Cancer Center, University of Illinois at Chicago, Chicago, IL 60612, USA; 3 Jesse Brown VA Medical Center, Chicago, IL 60612, USA

**Keywords:** cancer, transcription factors, oncogenes, therapeutic-resistance

## Abstract

Cancer is a multi-step process and requires constitutive expression/activation of transcription factors (TFs) for growth and survival. Many of the TFs reported so far are critical for carcinogenesis. These include pro-inflammatory TFs, hypoxia-inducible factors (HIFs), cell proliferation and epithelial–mesenchymal transition (EMT)-controlling TFs, pluripotency TFs upregulated in cancer stem-like cells, and the nuclear receptors (NRs). Some of those, including HIFs, Myc, ETS-1, and β-catenin, are multifunctional and may regulate multiple other TFs involved in various pro-oncogenic events, including proliferation, survival, metabolism, invasion, and metastasis. High expression of some TFs is also correlated with poor prognosis and chemoresistance, constituting a significant challenge in cancer treatment. Considering the pivotal role of TFs in cancer, there is an urgent need to develop strategies targeting them. Targeting TFs, in combination with other chemotherapeutics, could emerge as a better strategy to target cancer. So far, targeting NRs have shown promising results in improving survival. In this review, we provide a comprehensive overview of the TFs that play a central role in cancer progression, which could be potential therapeutic candidates for developing specific inhibitors. Here, we also discuss the efforts made to target some of those TFs, including NRs.

## 1. Introduction

Cancer is defined as the uncontrolled proliferation of cells caused primarily by dysregulation of the proto-oncogenes and/or tumor-suppressor genes. Thus, a proto-oncogene may become an activated “gain-of-function” oncogene due to mutation, chromosomal translocation, gene amplification, and epigenetic changes. Similarly, tumor-suppressor genes, the negative regulators of the cell cycle, may become inactivated due to “loss of function” mutations or deletion in one of the alleles. Oncogenes may be categorized as intracellular signal transducers, cell cycle regulators, growth factors or growth factor receptors, inhibitors of apoptosis, and transcription factors (TFs). It is estimated that about 20% of the oncogenes are identified as TFs [1]. In this review, we focus on understanding the function of some of those crucial TFs. Cancer cells require the constitutive expression of these TFs to sustain various biological processes that support their growth and survival for cellular transformation. On the other hand, the tumor-suppressor TFs are the negative regulators of cell cycle and are involved in DNA repair and apoptosis. Loss of function of tumor-suppressor TFs leads to uncontrolled cell division and progression of cancer [2]. p53 is the most studied tumor-suppressor TF that controls several genes involved in the induction of cell cycle arrest and apoptosis [3]. Not surprisingly, about 50% of the cancers show loss of function mutation in p53 gene [4], which is considered an important event in cancer progression. Tumor-suppressor TFs also act in suppressing metastasis. For instance, Krüppel-like factor 4 (KLF4) maintains the expression of E-cadherin in breast cancer cells [5], and reduces the expression of Slug in prostate cancer [6] to suppress metastasis. Similarly, induction of KLF4 is associated with reduced stemness properties, suppression of migration and invasion, and a mesenchymal-epithelial transition (MET) in nasopharygeal carcinoma [7]. DPC4 and MADR2 are other two tumor-suppressor TFs which are activated by TGFβ to inhibit cell proliferation and are inactivated in pancreatic adenocarcinomas and colon cancer, respectively [8,9,10]. WT1, Wilms tumor-suppressor TF suppressed the transcription of growth-promoting genes and was found to be less expressed in breast cancer [11]. As an additional complexity, a recent study has identified genes with paradoxical functions through database searches and indicated that 50% of those genes were for TFs which can have both oncogenic and tumor-suppressor functions in different cancers [12].

**Figure 1:**
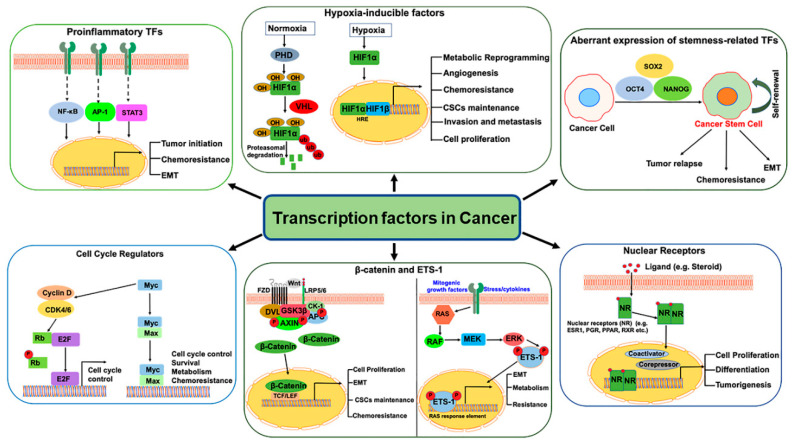
Schematic diagram representing the transcription factors activated in cancer to regulate the expression of genes involved in tumor growth, metastasis, chemoresistance, epithelial–mesenchymal transition (EMT), metabolism and Cancer Stem Cells (CSCs) maintenance.

TFs are proteins with DNA binding domains that bind to the specific sequences of DNA in the promoter and/or enhancer region and interact with other cofactors and RNA polymerase II to regulate target gene transcription. Various direct and indirect mechanisms can alter TF activity in cancers. The direct mechanisms include chromosomal translocations, gene amplification or deletion, point mutations, and alteration of expression, whereas non-coding DNA mutations, epigenetic mechanisms such as DNA methylation and histone modification are some of the indirect mechanisms [13]. TFs are the mainstay of cancer due to their crucial role in the initiation/progression of cancer as well as in invasion, metastasis, and chemo-resistance (Figure 1). Despite being considered “undruggable,” a better understanding of TF regulation (expression, degradation, protein/protein interaction) and the dynamics of their mode of DNA binding has changed this hypothesis and opened new possibilities to utilize TFs as cancer drug targets. In this review, we discuss some of the crucial oncogenic TFs which have the potential to be effective therapeutic targets to combat cancer. The expression and/or activity of these oncogenic TFs are often induced in cancer, thus increasing their druggability potential, compared to the low-expressing (undruggable) ones. Most of the current therapeutic options available or under-study are focused on targeting these oncogenic TFs.

## 2. Pro-Inflammatory Transcription Factors

Chronic inflammation caused by chemical and physical agents [14] and chronic virus infection such as human papillomavirus in cervical cancer [15] and hepatitis B and C in hepatocellular carcinoma (HCC) [16,17] increases the risk of malignancy. Cytokines produced by the inflammatory cells activate key TFs, such as NF-kB, STAT3, and AP1, involved in the dynamic regulation of hundreds of genes to control numerous pro-tumorigenic processes, including proliferation, survival, growth, angiogenesis, and invasion as discussed below.

### 2.1. Nuclear Factor-kappa B (NF-kB)

NF-κB is required for tumor initiation and suppression of immune surveillance of both innate and adaptive immune cells [18]. Its constitutive expression has been associated with increased cell proliferation, survival, invasion, and metastasis [19]. NF-kB inhibits programmed cell death via promoting transcription of genes that can block apoptosis by TNF-α and others [20], or by inducing anti-apoptotic factors, which include Bcl-xL [21], Survivin [22] and cellular inhibitors of apoptosis (cIAP-2) [23]. Moreover, NF-kB stimulates the transcription of Cyclin D1, a key regulator of G1 checkpoint control of cell cycle, thus, contributing to cancer progression [24].

### 2.2. Activator Protein 1 (AP-1)

The release of pro-inflammatory cytokines, growth factors, increase in oxidative stress, and treatment with other tumor-promoting substances are some of the well-established mechanisms that result in the activation of AP-1 [25,26]. It can promote both apoptosis and survival in a context-dependent manner. For example, AP1 target genes FasL, Bim, or Bcl3 could be either positive or negative regulators of apoptosis, and the balance between the pro- and anti-apoptotic target genes determines the final outcome of AP1, whether the cell will survive or undergo death [27,28]. Among the family members of AP-1, c-jun is involved in proliferation and survival, while JunB has the opposite effect. Moreover, it has been reported that c-jun regulates nearly a third of the TNFα-regulated transcriptome in triple-negative breast cancer (TNBC) [29] and likely facilitates tumor progression and metastasis. 

### 2.3. Signal Transducer and Activator of Transcription 3 (STAT3)

STAT3 plays a very crucial role in the initiation and progression of cancer by promoting a pro-carcinogenic inflammatory microenvironment [30,31], while suppressing anti-tumor immunity [32]. Several inflammatory factors encoded by NF-κB target genes, specifically interleukin-6 (IL-6), are important STAT3 activators [22,33]. IL-6-mediated STAT3 activation is reported to promote liver cancer [34]. Several infectious agents depend on STAT3 activation to cause inflammation-induced cancer; for example, H. pylori-induced gastric cancer [35]. Many tumor viruses such as hepatitis B virus [36], human papillomavirus [37], human T-lymphotropic virus type 1 [38], Epstein–Barr virus [39] also activate STAT3 by distinct mechanisms. STAT3 is involved in various stages of pancreatic cancer, and given its role in driving PDAC progression, it is considered an important therapeutic target [40].

## 3. Hypoxic Tumor Microenvironment in Cancer

### 3.1. Hypoxia-Inducible Factors (HIFs)

In most solid tumors, the rapidly growing cells generate regions of hypoxia because of aberrant vascularization and inadequate oxygen [41], which often lead to more aggressive tumor behaviors [42,43], therapy resistance and poor prognosis [44,45]. To cope with the hypoxic condition, tumor cells express hypoxia-inducible factor-1α (HIF-1α) as well as HIF-2α and HIF-3α [46,47]. Under hypoxic conditions, HIF-1 binds to the hypoxia-response elements (HREs) and induce the expression of numerous hypoxia-responsive genes involved in angiogenesis, metabolic adaptation, survival, and migration [48]. HIF-1α induces expression of glycolytic enzymes such as hexokinases and phosphoglycerate kinase 1 [49] and simultaneously stimulates glucose uptake by upregulating glucose transporters, GLUT1 and GLUT3 [50,51,52]. GLUT1 expression represents more aggressive tumors and is suggested to be an ideal prognostic cancer biomarker [53]. Increased glucose consumption by cancer cells produces high levels of lactic acid, which is expelled into the extracellular fluid through proton-linked monocarboxylate transporters (MCT4), also upregulated by HIF-1α [54]. Lactic acid accumulation leads to acidification of the tumor microenvironment (TME), leading to tumor progression, increased invasion, impaired immune functions [55,56,57], and impairment of drug uptake by cancer cells [55,58] resulting in treatment failure. 

### 3.2. HIFs in Cancer Stem Cells (CSCs)

A number of studies have highlighted the heterogeneous tumorigenic potential of cancer cells, which led to the discovery of CSCs [59,60]. They reside within the hypoxic sites of tumors [61] and can invade and metastasize to the distant places to re-initiate a secondary tumor. The self-renewal and stemness properties of the CSCs are controlled by the same pathways, including Notch, Hedgehog and Wnt, and TFs such as OCT4, SOX2, and NANOG, as in embryonic stem (ES) cells [62]. The induction of stemness TFs in cancers defines poor clinical outcomes and treatment resistance [63], promoting lineage plasticity leading to chemoresistance [64,65]. Both HIF-1α and HIF-2α are essential for CSC maintenance [66,67] and are required for different functions. HIF-1α is associated with the survival of the CSCs [68,69], and for the acquisition of chemoresistance [70]. On the other hand, HIF-2α is mostly required for the maintenance of stemness properties in CSCs, by upregulation of stemness TFs such as Oct4 [71,72]. Forced expression of non-degradable HIF2α is suggested to induce NANOG, OCT4, and c-Myc in non-CSCs in glioblastoma to promote survival and metastasis [73]. 

## 4. TFs Regulating Cell Proliferation, Invasion, and Metastasis

### 4.1. Myc

The Myc family of TFs contains three highly related genes, c-Myc, N-Myc, and L-Myc, which are well known to have their roles in transformation, cell proliferation, growth, differentiation, and apoptosis [74]. Although the expression of Myc is tightly regulated in normal cells, it is frequently deregulated in cancers [75]. Mutation of upstream signaling pathways, chromosomal rearrangements, insertion of retroviral promoter and enhancer, activation of super-enhancers within its own gene, can lead to the induction of Myc expression in cancer [76]. Evidence indicates that c-Myc can function to stimulate elongation factor and CTD of RNA Polymerase II at certain genes in the tumor [77,78] to increase the expression of most of the target genes [79]. c-Myc stimulates cell cycle progression by increasing transcription of positive cell cycle regulators such as cyclin-dependent kinases (Cdks), cyclins, and E2F TFs [80,81] and inhibiting transcription of the negative regulators such as p21 and p27 [82,83]. Overexpression of Myc can reprogram the cancer cells towards a stem-cell-like phenotype to favor tumor initiation, progression, and chemoresistance [84,85]. Myc-transformed cancer cells also showed profound metabolic changes as demonstrated by increased utilization of glucose and glutamine and increased expression of important glycolytic and glutaminolytic enzymes [86,87,88].

### 4.2. E2F

E2Fs are another family of TFs deregulated and hyperactivated in cancer cells. The binding of pRB to E2F inhibits its activity and prevents the G1-S phase of cell cycle transition. In the presence of growth factors, the cell cycle-dependent kinase complexes become activated sequentially, starting with Cdk4/Cdk6–cyclin D, followed by Cdk2–cyclin E. These activated CDKs then phosphorylate pRB proteins, resulting in the release of E2F and subsequent G1-S- transition [89,90]. The high E2F transcriptional activity observed in most cancers is because of the inactivation of pRB and overexpression of CDKs or inactivation of CDK inhibitors [91,92]. Among all the members of E2F, deregulation of E2F1 has been found in many cancer types [93,94,95]. In normal cells, E2F can induce apoptosis through induction of several pro-apoptotic genes such as Arf, p73, BH-3-only proteins, caspases and p53 related pro-apoptotic cofactors such as ASPP1 and ASPP2 to maintain hemostasis [96]. Many survival pathways, such as the Ras/MAPK pathway, PI3K/AKT pathway, Notch/Wnt/Hedgehog pathways [97,98], are constitutively activated in cancer cells and could be responsible for overcoming the apoptotic function of E2F in cancer. 

### 4.3. ETS1

E26 transformation-specific (ETS) are a family of 28 transcription factors, all of which contain a highly conserved ETS domain for DNA binding, and many of which are involved in cancer [99]. ETS TFs are considered as major oncodrivers, linking family members FL1 in Ewing sarcoma, ETS in leukemia [100], and ERG gene fusion in prostate cancer [101]. The founding member of the family ETS1 can be phosphorylated by MAPK, which increases its transcriptional activity [102]. ETS1 can induce transcription of the downstream target genes [103], many of which are involved in cell proliferation, survival, invasion, and angiogenesis [104,105,106]. ETS1 is associated with poor prognosis and therapy resistance in most of the cancers [107,108,109,110]. ETS1 is required for ZEB2-induced EMT phenotype, where it regulates ZEB2-mediated expression of Twist and MMP-9 [111], and can function as a master regulator of TGF-β-associated EMT in the mesenchymal subtypes of head and neck squamous cell carcinoma (HNSCC) [112]. ETS1 has been shown to regulate cellular metabolism in ovarian cancer cells resulting in the up-regulation of key enzymes involved in glycolysis [113]. Although ETS1 can be transcriptionally induced by reactive oxygen species (ROS) [114], it increases intracellular glutathione (GSH) levels via inducing the activity of glutathione peroxidase enzymes (GPX) to protect cancer cells from oxidative stress [115].

### 4.4. β-Catenin

β-catenin is a multifunctional protein that associates with the cytoplasmic region of E-cadherin to regulate homotypic cell-cell adhesion [116]. It also participates in the canonical Wnt signaling pathway by acting as a co-activator of the Lymphoid Enhancer Factor/T-Cell Factor (LEF/TCF) family of TFs to induce target gene transcription [117]. The activation of Wnt/β-catenin signaling in cancer plays a central role in the cell cycle and contributes to the neoplastic transformation through two of its main downstream targets, c-Myc and Cyclin D1 [118]. β-catenin is also suggested to protect the cancer cells from chemotherapy-induced apoptosis [119]. Microarray analysis of β-catenin knockdown cells revealed significant alteration in the expression of 130 genes involved in important cell-apoptotic pathways including, PTEN-PI3K-AKT, NF-κB, and p53 pathways [120]. During EMT, downregulation of E-cadherin leads to nuclear translocation of β-catenin, which increases β-catenin/TCF4 transcriptional activity [121] to induce the expression of ZEB1 and increases EMT and invasion [122]. The activation of ZEB1 further downregulates E-cadherin expression, thus sustaining activation of the positive feedback loop [123]. Wnt/β-catenin signaling pathway also plays a vital role in maintaining CSCs by controlling the expression of CSC markers and the pluripotency genes [124,125]. The inhibition of β-catenin is suggested to reduce CSC phenotype and tumor progression [126,127].

## 5. Nuclear Receptors (NRs)

NRs are a family of ligand-regulated TFs that possess a DNA-binding domain and a C-terminal ligand-binding domain that interacts with the ligands such as estrogen and progesterone to regulate the transcription in some hormone-sensitive tumors including breast, ovarian and prostate cancer [128,129]. Estrogen receptor alpha (ERα) and progesterone receptors (PR) are the examples of NRs, which bind to estrogen and progesterone, respectively, and regulate the transcription of downstream hormone-responsive genes in the target tissue [130,131]. Approximately 70% of breast cancers express either ERα, PR, or both and are considered hormone receptor-positive (HR+) [132]. ERα is well known to promote EMT and metastasis in breast cancer [133]. NRs recruit coactivator complexes to target genes for efficient transcriptional regulation [134]. Dysregulation of ER coregulators such as Amplified-in-breast cancer 1 (AIB1/NCOA3) and steroid receptor coactivator-1 (SRC-1) in breast cancer promotes invasion and metastasis [135,136]. The expression of ERα determines the phenotype of breast cancers and is one of the most important therapeutic targets. Likewise, PRs also function as critical regulators of transcription in breast cancer and are known to activate several signaling pathways, many of which are associated with the proliferation of breast cancer [137]. Several different types of NRs have been described so far, and details about these have been included under their respective inhibitor sections.

## 6. Chemoresistance and TFs

Chemotherapeutic drugs used for the treatment of cancers are effective for targeting the tumor initially. However, later the tumor develops resistance to evade the effects of chemotherapy, which leads to treatment failure. The molecular mechanisms by which tumors develop chemoresistance could be either intrinsic or acquired. Intrinsic resistance depends on the cancer cell’s intrinsic ability, such as DNA-damage repair, inactivation of drugs, and alteration of the drug targets. Acquired resistance develops during the course of treatment through the acquisition of different mechanisms such as the enrichment of CSCs, EMT, inhibition of apoptosis, increased drug transporter pumps, and mitochondrial alterations [138,139]. Aberrant expression and constitutive activation of TFs in cancers activate many of those genes involved in chemo-resistance (summarized in Table 1). 

## 7. Targeting TFs in Cancers

Considering the pivotal role of TFs in cancer, significant efforts have been made towards developing TF-targeting drugs. Different approaches, including targeting NR ligand-binding domains, essential protein–protein interactions, modulating proteasomal degradation of TFs, disrupting TFs-DNA binding, targeting activity by modulation of post-translational modifications, have been utilized [140]. A number of drugs targeting TFs in cancer are currently under different phases of clinical trials. Here, we discuss some of those (Table 2).

### 7.1. STAT3 and NF-κB Inhibitors

Most of the inhibitors currently in clinical trials for the inhibition of STAT3 are the small molecule inhibitors, including OPB-31121, OPB-51602, TTI-101, and WP1066, which prevent the phosphorylation and activation of STAT3 pathways. Another STAT3 inhibitor, which is in a clinical trial and has promising results, is an antisense oligonucleotide, AZD9150 (NCT01563302), with potential antitumor activity [141]. Most of the phytochemicals are also shown to inhibit STAT3, NF-κB, and AP1 [142]. Considering that several phytochemicals such as curcumin, and diindolylmethane (DIM), a bioactive metabolite of indole-3-carbinol found in cruciferous vegetables, are being investigated in phase I and phase II clinical trials for different cancers. IMX-110, a water-soluble formulation composed of nanoparticles encapsulating a STAT3, NF-κB, poly-tyrosine kinase inhibitor (TKI), and doxorubicin is also under phase I and II clinical investigation for advanced solid tumors including, pancreatic cancer, breast cancer and ovarian cancer (NCT03382340).

### 7.2. Myc Inhibitors

BRD4, a member of the bromodomain and Extraterminal (BET) protein family, is a transcriptional and epigenetic regulator playing an essential role during embryogenesis and cancer development [143]. BRD4 interacts with hyperacetylated histone and recruits the transcription machinery to promote gene transcription [144]. BRD4 regulates transcription of Myc, which in turn is involved in the progression of a variety of cancers [145,146,147]. Therefore, targeting BRD4, is one of the strategies to target Myc and several BET inhibitors such as BMS-986158, RO6870810, and GSK525762 are in different phases of preclinical and clinical trials for the treatment of different cancers. Moreover, recent reports suggesting that BRD4-mediated regulation of EMT-TFs is required for the invasion and metastasis of cancer cells [148,149] further rationalize the future use of BRD4 as a promising therapeutic target for aggressive tumors. Aurora kinase A (AURKA), a family member of mitotic serine/threonine kinases, provides stability to Myc protein by directly binding to it and inhibition of this protein–protein interaction using novel AURKA inhibitor, MLN8237 has shown promising results in HCC [150,151]. This drug has also undergone a clinical trial. 

### 7.3. HIF1 Inhibitors

Considering the major role of HIFs in cancer, several HIF-inhibitors have been developed which are in clinical trials. They can inhibit the expression and/or functions of HIFs, through direct or indirect mechanisms involving targeting the upstream or downstream pathways affecting HIFs. Vorinostat, an HDAC inhibitor, promotes degradation of HIF-1 indirectly through HDAC inhibition and is in clinical use to treat cutaneous T cell lymphoma [152,153]. PT2385 is a first-in-class HIF-2 inhibitor that inhibits the dimerization of HIF-2 and is under clinical investigation for the treatment of advanced clear cell renal cell carcinoma. Inhibitors targeting the mRNA expression of HIF-1α, such as EZN-2208 [154] and RO7070179 (EZN-2968), are also under clinical trial. A phase I study of EZN-2208 (NCT01251926) in patients with neuroblastoma showed low toxicity and was associated with clinical benefits [155]. RO7070179 has been suggested to potentially benefit HCC patients [156]. Several drugs inhibiting HIF-1α protein synthesis such as Topotecan, CRLX101, a nanoparticle conjugate containing the payload camptothecin (CPT) are undergoing clinical investigation for HIF-overexpressing cancers (NCT01652079). Digoxin (a cardiac glycoside), used for treating congestive heart failure and arrhythmias, also inhibit HIF-1α synthesis and show anti-cancer effects [157] and is in phase II of a clinical trial.

### 7.4. E2F Inhibitors

The CDK-RB-E2F pathway plays a major role in the mammalian cell cycle regulation, and so, it is undoubtedly an ideal target for the development of chemotherapeutic agents [158,159]. The interruption of E2F binding to the specific DNA sequences on the target gene is one of the approaches used for targeting E2F-mediated transcription. Efforts to block the Cyclin D/CDK4/6 pathway required for the functioning of E2F have led to the discovery of CDK4/6 inhibitors [160]. CDK-inhibitors re-activate RB by preventing its phosphorylation and maintain efficient transcriptional repression. palbociclib, ribociclib, and abemaciclib are the small molecule CDK4/6 inhibitors currently approved for breast cancer treatment and are under clinical investigation for other solid tumors. A next-generation CDK4/6 inhibitor, G1T38, was developed to minimize the side effects of Palbociclib, which causes severe neutropenia [161], and was recently entered into phase I and II trials.

### 7.5. Wnt/β-Catenin Inhibitors

The interaction of Wnt-secreted proteins with LRP5/6 coreceptors and frizzled (FZD) receptors on the cell surface is the first step in Wnt/β -catenin signaling [162]. Disruption of this interaction is, therefore, used as an approach to target the Wnt/β-catenin pathway. Vantictumab is a first-in-class monoclonal antibody that interacts with FZD receptors 1, 2, 5, 7, and 8 and blocks Wnt signaling [163]. Ipafricept (OMP-54F28) is a recombinant fusion protein that combines the cysteine-rich domain of FZD receptor 8 with a human IgG1 Fc fragment, which competes with FZD8 receptor for its ligands and antagonizes Wnt signaling [164]. β-catenin recruits its coactivator CBP (cAMP-responsive element-binding protein) to promote target gene transcription [165]. PRI-724 (a second-generation compound of ICG-001) has emerged as a novel small molecule antagonist that binds to CBP to block β-catenin and CBP interaction, and is in the early phase of a clinical trial for advanced solid tumors (NCT01764477). CWP232291 is another potent inhibitor of Wnt/β-catenin-mediated transcriptional activity and is currently scheduled for phase I/II and phase I clinical trials in acute myeloid leukemia (NCT03055286), multiple myeloma (NCT02426723) respectively. Some members of the Wnt family, such as WNT5A, act as an antagonist of canonical Wnt signaling in some cancers such as colon cancer [166] breast cancer [167] and leukemia [168]. Therefore, using a small WNT5A-mimicking peptide Foxy5 has been suggested as a future anti-metastatic treatment strategy for breast cancer patients [169]. The drug is under phase II clinical investigation to treat colon cancer.

## 8. Targeting Druggable Nuclear Receptors

The NRs are known to contain a well-defined ligand-binding pocket and can be activated by natural ligands. This is why NRs are considered to be most druggable. The natural ligands that can activate NRs include retinoic acid, glucocorticoids, estrogen, androgens (testosterone and dihydrotestosterone), and long-chain fatty acids. The activities of NRs can be modulated by small molecules that mimic their natural ligands, thus making them ideal therapeutic targets. Of all the small-molecule drugs approved by the US Food and Drug Administration, about 14% are believed to target NRs [170]. Targeting NRs constitute a central aspect of many cancer therapies, as they show tumor-specific effects. In fact, the status of NR in cancer patients show important correlations with their survival and treatment modalities, as has been demonstrated in multiple studies [171]. Here, we provide an overview of selected NRs (ER, AR, GR, PR, RAR, and PPAR) currently being pursued as therapeutic targets in different types of cancer. We summarized the most recent drugs that are under clinical trials under phase I–IV for the last 1.5 years (Table 3).

### 8.1. Estrogen Receptor α Inhibitors

ER is one of the NRs which is always at the forefront of anticancer drug discovery. The two isoforms of ERs are ERα (NR3A1), encoded by *ESR1* and ERβ (NR3A2), encoded by the *ESR2* gene. ERα drives the pathogenesis of breast cancer in most of the patients [172]. ER-directed therapies are the foundation in the management of ER+ breast cancer. Tamoxifen was the first approved drug to inhibit ERα for reducing breast cancer risk and is known as a selective ER modulator (SERM) because of its selective effect on ERs in different tissues [173]. Later efforts made to identify inhibitors that can selectively target ERα in breast cancer tissue led to other NR-based drugs known as selective ER degraders (SERD). Fulvestrant (a SERD) is regarded as a pure ERα antagonist that is more efficacious than tamoxifen in suppressing breast cancer cell proliferation [174]. These endocrine therapies block the ER signaling pathway and are highly effective, but its usefulness is limited by common intrinsic and acquired resistance. For example, Forkhead box protein A1 (FOXA1) can alter ER transcriptome via IL-8 expression to promote endocrine resistance [175]. Studies also show that differential interactions of ER α and oncogenic TFs can result in reprogramming of enhancers, ultimately leading to endocrine therapy-resistance [176]. The second-generation SERM, raloxifene, was later approved, which has the advantage that it does not increase the incidence of primary coronary risk in postmenopausal women [177]. A number of new-generation SERMs are under clinical investigation. Bazedoxifene is a new chemical entity different from raloxifene or tamoxifen that binds with higher affinity with both ERα and ERβ. This drug does not cause hot flashes and also preserves bone density in postmenopausal women [178]. Since ERα is overexpressed and the *ESR1* gene is mutated in the ER+ cancer tissues, degradation of ER was considered an alternate and more specific treatment option. As mentioned above fulvestrant was the first identified SERD, which has been used in combination with CDK4/6 inhibitors, includingpalbociclib, abemaciclib and ribociclib and drugs that target PI3K/AKT/mTOR pathways such as pictilisib, vistusertib *(AZD2014)* and everolimus [179,180,181,182]. The orally bioavailable drug brilanestrant (GDC-0810) is a dual-function SERD that has the function of ER degradation and ER antagonism [183]. Another next-generation SERD elacestarnt (RAD1901) is orally bioavailable, can cross the blood-brain barrier, and showed potent growth inhibitory activity in several models of ER+ breast cancer [184]. Other ER inhibitors are summarized in Table 3 that either completed or are in ongoing clinical trials.

### 8.2. Androgen Receptor Inhibitors

Androgens, such as testosterone and dihydrotestosterone, mediate their functions via the androgen receptor (AR, NR3C4). ARs control the development of the prostate gland and prostate carcinogenesis [218]. Two strategies are currently available to target androgen signaling; the first is androgen deprivation therapy (ADT), and the second is antiandrogen treatment. Antiandrogens that are used for treating advanced prostate cancer patients are either steroidal or nonsteroidal (Nonsteroidal antiandrogens, NSAAs) in nature. Flutamide, bicalutamide, and nilutamide were the first-generation antiandrogens, followed by enzalutamide, which was developed as a second-generation compound. All of these compounds antagonize AR activity, resulting in inhibition of tumor growth [219]. Another second generation NSAA compound, apalutamide, that irreversibly binds with AR in the cytoplasm and inhibits nuclear translocation [220], has been approved for non-metastatic castration-resistance prostate cancer (CRPC). Darolutamide (also known as ODM-201) was discovered as a next-generation AR antagonist, which blocks AR translocation into the nucleus [221]. A phase III study concluded that in men with nonmetastatic-CRPC (nmCRPC), metastasis-free survival was significantly longer with darolutamide than with placebo [222]. Strategies were developed to impair AR signaling by pharmacologically degrading AR, designated as SARDs. Cytochrome P450 17A1 (CYP17A1) is a hydroxylase type of enzyme involved in the synthesis of AR ligands, dehydroepiandrosterone (DHEA), androstenedione, and testosterone. Abiraterone acetate inhibits CYP17, by inhibiting its C17, 20-lyase and 17α-hydroxylase activities. A recent ongoing phase II trial study is evaluating the combinatorial effect of apalutamide, abiraterone acetate, and other drugs in treating patients with advanced prostate cancer (NCT02849990) [223]. Selective AR modulators (SARMs) were also developed as nonsteroidal AR agonists. Enobosarm (GTx-024) was developed as a SARM, not to treat cancer, but for the prevention and treatment of cachexia [224]. A phase II study, with a combination of enobosarm and pembrolizumab (an immune checkpoint inhibitor), is currently evaluating its therapeutic efficacy in patients with AR+ metastatic TNBC (NCT02971761). Another compound, MK4541, was discovered as a SARM that could potentially induce caspase-3 activity and apoptosis in prostate cancer cells [225]. AR is also targeted for hormone receptor-positive breast cancer in postmenopausal women (NCT03088527). RAD140, a SARM is currently under phase I clinical trial, which can bind to and activate AR with high affinity and specificity in breast cancer cells but not in prostate cancer cells. 

### 8.3. Glucocorticoid Receptor (GR) Inhibitors

Glucocorticoids have been used to dampen the immune system. Glucocorticoids are often used during breast cancer chemotherapy to mitigate the allergic reaction [226]. In the cancer scenario, the beneficial anti-inflammatory effect of GR (NR3C1) is derived from the transrepression of GR target genes, while GR transactivation is undesirable. One mechanism to achieve transrepression is by preventing the formation of GR homodimers that negatively influence high-affinity binding to the glucocorticoid-responsive element (GRE) of the gene promoters [227]. Selective GR agonists and modulators (SEGRAMs) such as RU24858, 21OH-6, 19OP, avicin D, and compound A (CpdA) are some of the compounds that work through a conformational change in GR [228], potently transrepressing AP-1 and NF-κB pathways [229]. Avicin D and CpdA are plant-derived triterpenoid saponins with GR-mediated anti-inflammatory functions [230,231]. CpdA can induce apoptotic cell death in the prostate, bladder, leukemia, and multiple myeloma cell lines [231]. Table 3 includes the limited number of SEGRAMs that are in clinical trials for cancer treatment, one of which is relacorilant (CORT125134). Phase I and II clinical trials are currently underway with relacorilant in combination with nab-paclitaxel for the solid tumors [232]. Another highly potent GR antagonist ORIC-101, which is used for CRPC in AR+ tumors, has shown antitumor effects by promoting a response to chemotherapy in an ovarian cancer xenograft model [233].

### 8.4. Progesterone Receptor (PR) Inhibitors

Progesterone plays an important role in regulating normal female reproductive functions. Mifepristone (RU486) is an extensively studied modulator of PR (NR3C3), which also modulates GRs and ARs [234]. As PRs have significant functional importance in regulating the physiology of mammary glands, PR modulators are being investigated for their therapeutic potential towards breast cancer. A phase II clinical trial is currently underway with mifepristone in breast cancer patients (NCT02651844). Telapristone (CDB-4124) and onapristone (ZK-98299) are some of the other synthetic steroids that have been characterized for a long time [235]. Telapristone acetate, a selective PR modulator (SPRM), has been shown to inhibit precancerous lesions and carcinogen-induced ER+ breast tumors in rats [236]. Onapristone, a structurally similar compound as mifepristone, is a pure PR antagonist without any agonistic activity. It produced antitumor effects in various models of hormone-dependent mammary tumors [235]. Onapristone antagonized nuclear localization of phospho-PR (S294), and when used in combination with trametinib (a MEK/ERK dual kinase inhibitor), produced excellent effects in uterine cancer models [237]. Despite these encouraging results, a clinical trial with onapristone was discontinued, as some patients showed anomalies in liver function tests. Two more clinical trials were performed with onapristone in 2013, one for PR-expressing cancers (NCT02052128) and the other for advanced CRPC (NCT02049190). However, multiple clinical trials with SPRMs have shown either lack of reproducibility or undesired toxicity in ER+ breast cancer patients. Taken together, these study results indicate an urgent need for the development of newer-generation of SPRMs with significantly improved clinical efficacy and better safety profiles [238]. It is also important to note that there is a substantial interplay between PR and ERα. PR can regulate the binding of ERα to chromatin and modulate its transcriptional activity that correlate with a good clinical outcome. It is thus possible, that PR agonists can provide therapeutic benefits to those ERα+ breast cancer patients who show resistance to ERα antagonists [239].

### 8.5. Retinoic Acid Receptor Inhibitors 

Retinoic acid receptors (RARs) are ligand-dependent TFs that heterodimerize with retinoid X receptors (RXRs) to regulate various cellular functions, including cellular growth, differentiation, and apoptosis. Retinoids, which are precursors of vitamin A, function as the endogenous ligands for both RARs and RXRs, and are involved in mediating various physiological processes [240]. All-trans retinoic acid (ATRA) is believed to be the most predominant and ubiquitously-expressed natural ligand in the human body [241]. RARs include three isoforms, RARα (NR1B1), RARβ (NR1B2), and RARγ (NR1B3), and those of RXRs include RXRα (NR2B1), RXRβ (NR2B2), and RXRγ (NR2B3). RARs and RXRs can heterodimerize with each other in various combinations. RAR-RXR heterodimers bind to specific DNA sequences in the target gene promoters, known as retinoic acid response elements (RAREs), and regulate transcription [242]. However, RXRs can also heterodimerize with other NRs and modulate target gene expression [129]. Alitretinoin (9-cis-retinoic acid), which is chemically similar to tretinoin (ATRA), can bind to RARs and RXRs with high affinity, and regulate cellular proliferation and apoptosis [243]. It is, however, contraindicated in pregnant women, due to its teratogenic property [244]. 

Currently, there is one ongoing clinical trial to determine the re-sensitization of non-APL AML cells (a subtype of AML) to ATRA when combined with lysine-specific demethylase 1 (LSD 1) inhibitor tranylcypromine (NCT02273102). Bexarotene is an RXR specific ligand that shares some structural similarity with classic retinoids, commonly referred to as “rexinoids”, and was approved by the FDA for the treatment of Cutaneous T-cell lymphoma (CTCL) [245]. Its therapeutic effects were also investigated for NSCLC patients, which showed significantly improved survival in subgroups of patients who had triglyceride elevations [246]. Few clinical trials assessing the effects of bexarotene for solid tumors and hematological malignancies (NCT01578499 and NCT00615784) have also been completed. Tamibarotene (SY-1425 or Am80) is a second-generation receptor subtype-selective retinoid, significantly superior to ATRA that has shown potent antitumor activity against Acute Promyelocytic Leukemia [247,248]. IRX5183 is a RARα agonist, with good oral bioavailability and with potential antitumor effects. Its binding transactivates RARα responsive genes, which leads to the induction of cellular differentiation and apoptosis, inhibition of cell proliferation, and tumorigenesis [249]. Currently, IRX5183 is in phase I/II clinical trials for relapsed and refractory AML and high-risk Myelodysplastic Syndrome (NCT02749708). Another compound, IRX4204, is an RXRα agonist, which activates RXRs downstream target genes and induces apoptosis leading to tumor regression [250]. IRX4204 also promotes differentiation of CD4+ T lymphocytes into inducible regulatory T cells. It can cross the blood–brain barrier, thus making it a potential drug candidate for brain tumors. A phase I clinical trial is underway in patients with advanced NSCLC, who were previously treated, to evaluate the therapeutic effects of IRX4204 in combination with erlotinib (NCT02991651). Table 3 shows the recent drugs for oncological applications. Lately, drug discovery companies are actively researching the drugs that can target RAR/RXR heterodimers. This has led to the discovery of 4-(3-chloro-4-ethoxy-5-isopropoxybenzamido)-2-methylbenzoic acid [251], which might enter preclinical investigations for cancer in the near future. 

### 8.6. Peroxisome Proliferator-Activated Receptor (PPAR) Inhibitors

PPARs are known to play important functions in regulating glucose and lipid homeostasis, inflammation, proliferation, and differentiation. Due to their well-defined role in regulating various cellular functions, PPAR ligands have also been investigated for their potential as cancer therapeutics [252]. The three members of the PPAR family are PPARα (NR1C1), PPARβ/δ (NR1C2) and PPARγ (NR1C3) that upon binding with the respective ligands (such as long carbon chain fatty acids), heterodimerize with RXR on the promoter of the downstream target genes and exert their biological functions. PPARγ activation by thiazolidinediones (TZDs) and similar ligands have the potential to trigger antitumorigenic effects. This can lead to various cellular effects, including cell-cycle arrest, induction of apoptosis, suppression of angiogenesis, and modulation of stemness. In addition, they can co-operate with other signaling pathways and modulate various cellular functions. Efatutazone (inolitazone, CS-7017, RS-5444), a third-generation TZD and an oral agonist for PPARγ, was shown to inhibit ATC cell proliferation by upregulation of RhoB and p21, and to synergistically potentiate apoptosis in combination with paclitaxel [253,254]. Efatutazone has entered clinical trials for several kinds of solid tumors and advanced anaplastic thyroid cancer (NCT02249949 and NCT02152137). A phase II trial was conducted using another PPARγ ligand, pioglitazone hydrochloride for head and neck cancer in patients with oral leukoplakia. The study concluded that pioglitazone hydrochloride might be effective in preventing head and neck cancer (NCT00099021). Iloprost is a dual PPARα/δ agonist, which has undergone a randomized phase II clinical trial. This study concluded that oral Iloprost could significantly improve endobronchial histology in former smokers. Further studies are warranted to determine its efficacy in preventing lung cancer development [255].

## 9. Conclusions

Cancer cells highly depend on the constitutive expression of TFs to support their growth and survival. Increasing knowledge of the mechanism of action and regulatory networks of the TFs has led to a better understanding of their roles in cancer and other diseases. Some of the critical TFs involved in carcinogenesis are inflammatory TFs such as NF-kB, STAT3, and AP1, which regulate genes for tumor initiation and progression. HIFs are required for the progression of cancer through the activation of genes which maintain CSCs, promote invasion, metastasis, growth, and angiogenesis. c-Myc and E2F1 deregulate the cell-cycle leading to uncontrolled cell division. β-catenin and ETS1 promote EMT and metastasis, while NRs play a pivotal role in hormone-sensitive cancers. Aberrant expression of many of these TFs after chemotherapeutic treatments leads to the acquisition of EMT and cancer stemness. These events foster chemoresistance and pose a significant challenge in making cancer treatment successful. Considering their importance in cancer, significant efforts have been made to develop drugs targeting TFs. Several of them have undergone clinical trials, but only a few succeeded in advancing to clinical use because of side effects, toxicity, and low tolerance. However, recent advancements in designing and development of drugs, including computer-aided molecular modeling and structure-based drug designs, have led to the development of better drugs to target TFs with minimal side effects. Disrupting protein–protein interactions and their binding to DNA, and restricting binding at the epigenetic level by modulating chromatin accessibility are the emerging new strategies to target TFs. So far, significant progress has been made to target NRs, and several NR-targeting drugs are FDA approved and currently in clinical use. Traditionally, the development of drugs targeting NRs was based on identifying the small molecules that mimic their natural ligands and can induce or repress their activity by binding to the ligand-binding domain in NRs. However, the acquisition of resistance in tumors against those drugs has led to the development of improved strategies to target NRs. These include targeting the NR DNA-binding domain and the DNA sites recognized by NRs. For a long period of time, TFs were considered as non-druggable targets. However, the emerging new drugs have given hope that targeting TFs could be achievable. Considering the dependency of cancer cells on TFs to acquire chemoresistance and the major obstacles with monotherapy, it is conceivable that a combination of drugs targeting TFs with the currently available chemotherapeutics could be an effective approach in cancer treatment in the future. Furthermore, since recent studies have suggested that NR-based therapeutics could also impact the immune response, TF-targeting drugs in combination with immunotherapies seem to offer great potential for long-term cancer treatment. Genomic profiling has revealed the intricacy between NRs and how they crosstalk to regulate sets of genes in cancer. Hence, in the future, higher throughput sequencing data of tumors will provide a new dimension to NR-based drug development.

In addition to the crucial TFs discussed in the review, there are others such as KLF5, FOXM1, GLI1, RUNX1, FOXO, and NRF2, which are also known to promote oncogenic activities. Different studies suggested that these TFs can also be the effective targets for anti-cancer therapies, and a few drugs are in clinical trials as well. However, the detailed mechanisms explaining the role of these TFs in the progression of different cancers are still unclear and need to be addressed before designing novel therapeutic drugs to target them effectively in cancer.

With all this information, it is crucial to be aware of the fact that TF expression is cell-specific, and in pathological conditions, the expression of subsets of TFs becomes more cancer-specific, so the development of TF inhibitors is not straightforward. Despite well-defined hallmarks of cancer in general [256], many cancers present themselves with specific deregulations that characterize the hallmarks for specific subtypes of cancer [257,258,259,260,261]. As TF profiles change with the landscape of cancer, a more tailored approach will be necessary to target TFs in specific cancer types. TFs regulate a plethora of genes that are also important for the normal physiological function of cells, thus adding more complexity and difficulty in targeting them specifically towards malignant cells. Given the fact that all cells require transcription for survival and maintenance, inhibitors targeting TFs may turn out to be toxic, and with unavoidable side effects. However, with the recent advances in techniques, the context-specific reliance of cancer cells on TFs is currently being exploited for the identification of a large number of therapeutic opportunities. Recent advances in functional genomics such as CRISPR-based genetic screens can provide systematic genetic analysis of TF dependencies across diverse forms of cancer [262]. In another approach, genome-scale RNAi-based loss-of-function screens were used to identify genes essential for cancer cell proliferation and survival [263]. These specific TF genes were nominated by the cancer dependency map project (DepMap) [264]. Generating chemical probes for these TFs might be key in the development of therapeutic agents with fewer side effects, due to enhanced specificity for the cancer cells.

## Figures and Tables

**Table 1:** Transcription factors in chemo-resistance.

Transcription Factors	Chemo-Resistance Mechanism	Cancer Type	Drug	Reference
NF-κB	p50 subunit of NF-κB associate with BRCA1 on the promoter of genes encoding anti-apoptotic proteins to promote BRC1-mediated resistance to DNA damage	Breast cancer	Etoposide and Camptothecin	[185]
	p65/RelA activation in 267B1/K-ras overexpressing tumorigenic cells promote chemo-resistance	Prostate cancer	Trichostatin A	[186]
	NF-κB regulates MDR1 gene expression	Colon cancer	Daunomycin	[187]
AP1	The AP-1 family member JunB promoted growth and dexamethasone-resistance in multiple myeloma cells. These were reversed by the silencing of JunB.	Multiple myeloma cell	Dexamethasone	[188]
	c-Jun upregulated FoxM1 in sorafenib-resistant liver cancer cells. Knocking down c-jun expression reversed this, resulting in enhanced sensitivity of cells to sorafenib.	Liver cancer	Sorafenib	[189]
	Activation of AP-1 expression induced transcription of XIAP conferring resistance to chemotherapeutics	Breast and liver cancer cells	HDAC inhibitor, JNJ-2648158	[190]
STAT3	Expression of pSTAT3 was higher in cisplatin-resistant cells and silencing of STAT3 increased chemotherapy sensitivity	Ovarian cancer	Cisplatin	[191]
	Activation of STAT3 in association with p53/RAS pathway controls metastasis and cisplatin-resistance. This also involves Slug, MAPK and PI3K/AKT axes.	Ovarian cancer	Cisplatin	[192]
	ID1 mediates resistance via STAT3-mediated induction of ATF6 transcription to induce autophagy	Ovarian cancer	Paclitaxel and cisplatin	[193]
	Involvement of Src/STAT3 signaling pathway in chemotherapy resistance	Triple negative breast cancer	Camptothecin, Doxorubicin	[194]
HIF-1	HIF-1 induced carbonic anhydrase IX expression. Inhibition of carbonic anhydrase IX restored chemo-sensitivity against vinorelbine	Lung cancer	Vinorelbine	[195]
	HIF-2α overexpression increased the expression of stem cell markers (c-Myc, OCT4, Nanog) and Paclitaxel-resistance. These were accomplished via activation of Wnt and Notch pathways.	Breast cancer	Paclitaxel	[196]
	Treatment of breast cancer cell lines with Paclitaxel or Gemcitabine increased HIF activity. This in turn enriched the cancer stem cell population and increased the expression of multidrug resistance 1 (MDR1)	Breast cancer	Paclitaxel or Gemcitabine	[70]
	HIF-dependent BMX kinase upregulation resulted in therapeutic resistance through a compensatory pro-survival signaling mechanism	Acute myeloid leukemia	Sorafenib	[197]
	HIF2 and COX2 activated Snail and downregulated E-cadherin expression to promote invasion and resistance to sorafenib	Renal cancer	Sorafenib	[198]
	HIF-2α activated TGF-α/EGFR pathway to promote proliferation and sorafenib-resistance, which was antagonized by HIF-2α siRNA	Hepatocellular carcinoma	Sorafenib	[199]
MYC	MYC cooperated with MCL1 to increase mitochondrial oxidative phosphorylation and ROS to maintain CSCs and promote chemo-resistance	Triple negative breast cancer	Paclitaxel	[200]
	c-Myc promoted the self-renewal, tumorigenicity, invasion and drug-resistance of colon CSCs	Colon cancer	5-Fluorouracil, Oxaliplatin, FOLFOX	[201]
	c-Myc upregulated tongue cancer resistance-associated protein 1 (TCRP1) to promote chemoresistance, and this axis acted as a negative biomarker of prognosis	Tongue and lung cancer	Cisplatin	[202]
	Overexpression of c-Myc promoted resistance to chemotherapeutic drugs, increased colony formation and inhibited cell differentiation	Acute Myeloid Leukemia Cells	Cytarabine (Ara-C), Daunomycin, Doxorubicin	[203]
ETS1	ETS1 upregulated MDR1 and MMP9 expressions to promote paclitaxel-resistance and invasion	Hormone-refractory prostate cancer	Paclitaxel	[204]
	ETS1 promoted cisplatin-resistance by transcriptional activation of genes involved in reducing cisplatin toxicity, including metallothioneins and DNA repair enzymes	Ovarian cancer	Cisplatin	[205]
	ETS1 binding to Pregnane X receptor (PXR) increases the transcriptional activity of PXR, leading to sorafenib-resistance through induction of multi-drug resistance genes	Hepatocellular carcinoma	Sorafenib	[109]
	ETS1 increased MDR1 expression, and siRNA-mediated silencing of ETS1 reduced MDR1 expression and effectively reversed drug-resistance	Adriamycin-resistant breast cancer cells	Adriamycin	[206]
β-catenin/TCF	Leucine-rich-repeat-containing G protein-coupled receptor (LGR) promoted stemness and chemo-resistance via activating Wnt/β-catenin signaling pathway	Ovarian cancer	Cisplatin and Paclitaxel	[207]
	β-catenin promoted survival of metastatic melanoma cells and not benign melanocytes or primary, non-invasive melanoma cells. Downregulation of β-catenin sensitized metastatic melanoma cells towards chemotherapy	Metastatic melanoma cells	Temozolomide, Cisplatin and Doxorubicin	[208]
	Prospero-related homeobox 1 (PROX1) upregulated β-catenin transcription and nuclear translocation to activate the Wnt/β-catenin pathway in HCC, which lead to high proliferation and sorafenib-resistance	Hepatocellular carcinoma	Sorafenib	[209]
	Nek2 stabilized β-catenin, increased its nuclear translocation, and activated the transcription of its downstream target genes, to promote sorafenib-resistance	Hepatocellular carcinoma	Sorafenib	[210]
	Suppression of checkpoint kinase 1 (CHK1) pathway by Wnt/β-catenin in p53 wild-type colorectal cancer cells, promoted drug-resistance	Colorectal cancer	5-Fluorouracil	[211]
Transcription factors related to stemness (Oct-4, Sox-2, Nanog)	Chemotherapy-induced Oct-4 expression promoted acquired resistance in cancer	Bladder cancer	Cisplatin	[212]
	High expression of Oct-4 in CD133+CSCs maintained self-renewal and drug-resistance in lung cancer. Knocking down Oct-4 expression in CD133+CSCs significantly inhibited tumor invasion and colony formation, and increased apoptosis	Lung cancer	Cisplatin, Etoposide, Doxorubicin, and Paclitaxel	[213]
	HDAC11-mediated increase in expression of Sox2 is required for the maintenance of CSCs to promote drug resistance	Lung adenocarcinoma	Cisplatin, Erlotinib and Gefitinib	[214]
	IL-6/p-STAT3 activation increased the expression of DNMT3b/OCT4 which conferred early recurrence and poor prognosis in HCC	Hepatocellular carcinoma	Sorafenib	[215]
	The expression of Sox2 and CD24 were upregulated in targeted-therapy resistant melanoma cells, which was mediated by activated STAT3. Activation of STAT3, Sox2 and CD24 promoted adaptive-resistance to BRAF inhibitors.	Melanoma	BRAF inhibitors (Vemurafenib, plx8394 and pIx7904)	[216]
	Increased expression of Oct-4 and Nanog play important roles in the proliferation, migration, invasion and chemoresistance of pancreatic CSCs, and might serve as important prognostic biomarkers and therapeutic targets for pancreatic cancer.	Pancreatic cancer	Gemcitabine	[217]

**Table 2:** Selected Inhibitors against Transcription Factors (TFs) in Clinical Trials.

TFs	Therapeutics	Phase	Cancer Type	Status	Trial Identifier
STAT3	OPB-31121	Phase I	Advanced solid tumors	Completed	NCT00955812
	OPB-51602	Phase I	Advanced cancers	Completed	NCT01423903
	AZD9150 (IONIS-STAT3Rx)	Phase I/II	Advanced cancers, Diffuse Large B Cell Lymphoma, Lymphoma	Completed	NCT01563302
	WP1066	Phase I	Metastatic malignant neoplasm in the brain, metastatic melanoma, recurrent brain neoplasm, recurrent glioblastoma, recurrent malignant glioma	Recruiting	NCT01904123
	TTI-101	Phase I	Breast cancer, head and neck squamous cell carcinoma, Non-small cell lung cancer (NSCLC), HCC, colorectal cancer, gastric adenocarcinoma, melanoma, advanced cancer	Recruiting	NCT03195699
NF-κB	Imx-110	Phase I/II	Advanced solid tumors, pancreatic cancer, breast Cancer, ovarian cancer	Recruiting	NCT03382340
	BR-DIM	Phase I	Nonmetastatic hormone-refractory prostate cancer	Completed	NCT00305747
	Curcumin	Phase II	Breast cancer	Completed	NCT01740323
HIF-1	Digoxin	Phase II	Breast cancer	Completed	NCT01763931
	Topotecan	Phase I	Refractory advanced solid neoplasms expressing HIF-1α	Completed	NCT00117013
	PX-478	Phase I	Advanced solid tumors, lymphoma	Completed	NCT00522652
	EZN-2208	Phase I	Refractory solid tumors (in combination with Bevacizumab)	Completed	NCT01251926
	CRLX101	Phase II	Recurrent platinum-resistant ovarian cancer, Fallopian tube cancer, primary peritoneal cancer (in combination with Bevacizumab)	Completed	NCT01652079
	RO7070179	Phase I	Hepatocellular carcinoma	Completed	NCT02564614
	Vorinostat	Phase I	Advanced Breast Cancer (in combination with Capecitabine)	Completed	NCT00719875
	PT2385	Phase I	Advanced clear cell renal cell carcinoma, kidney cancer (alone or in combination with nivolumab or cabozantinib)	Active	NCT02293980
Myc	BMS-986158	Phase I	Pediatric solid tumors, lymphoma, or brain tumor	Recruiting	NCT03936465
	GSK525762	Phase I/II	Relapsed, refractory hematological malignancies	Completed	NCT01943851
	MLN8237	Phase II	Histologically confirmed or clinically suspected metastatic neuroendocrine Prostate cancer	Completed	NCT01799278
	RO6870810	Phase I	Relapsed/refractory acute myeloid leukemia (AML)	Completed	NCT02308761
Wnt/Beta-catenin	PRI-724	Phase I	Pancreatic adenocarcinoma, which is locally advanced, metastatic, or otherwise inoperable. These patients are candidates for second-line therapy after failing FOLFIRINOX as first-line therapy (in combination with gemcitabine)	Completed	NCT01764477
	CWP232291	Phase I/II	Relapsed or refractory AML (in combination with Cytarabine)	Active	NCT03055286
	Vantictumab	Phase I	Previously untreated stage IV pancreatic cancer (in combination with nab-paclitaxel and gemcitabine)	Completed	NCT02005315
	Ipafricept (OMP-54F28)	Phase I	Solid tumors	Completed	NCT01608867
	Ipafricept (OMP-54F28)	Phase I	Previously untreated stage IV pancreatic cancer (in combination with nab-paclitaxel and gemcitabine)	Completed	NCT02050178
	Foxy-5	Phase II	Resected colon cancer patients treated with FOLFOX chemotherapy regimen	Recruiting	NCT03883802
E2F	Ribociclib	Phase I	Recurrent Glioblastoma or anaplastic Glioma	Unknown	NCT02345824
	Palbociclib	Phase I/II	Advanced KRAS mutant NSCLC (in combination with MEK inhibitor Binimetinib)	Recruiting	NCT03170206
	Abemaciclib	Phase II	Chemo-refractory, Rb wild-type extensive Small-cell lung cancer	Recruiting	NCT04010357
	G1T38	Phase I/II	EGRF mutation-positive metastatic NSCLC (in combination with Osimertinib)	Active	NCT03455829

**Table 3:** Inhibitors against Nuclear Receptors in Clinical Trials.

Nuclear Receptors	Therapeutics	Phase	Cancer Type	Status	Trial Identifier
ER	Bazedoxifene	Phase II	Ductal Breast Carcinoma In Situ	Recruiting	NCT02694809
	Z-Endoxifen Hydrochloride	Phase I	Breast cancer	Active	NCT01327781
	Lasofoxifene	Phase II	Locally Advanced or Metastatic breast cancer	Recruiting	NCT03781063
	Acolbifene Hydrochloride	Phase II	Breast cancer	Completed	NCT00853996
	Goserelin	Phase I	Patients with Advanced ER+ (Her2 Negative) Breast cancer	Active	NCT02586675
	Elacestrant (RAD-1901)	Phase III	ER^+^/HER2^-^ advanced breast cancer (EMERALD)	Recruiting	NCT03778931
	AZD9496	Phase I	Postmenopausal women with ER+ HER2− primary breast cancer	Completed	NCT03236974
AR	Darolutamide (BAY1841788, ODM-201)	Phase III	Non-metastatic CRPC (nmCRPC)	Active	NCT02200614
	Darolutamide	Phase III	Metastatic Hormone Sensitive Prostate Cancer (mHSPC) (in combination with ADT and Docetaxel)	Active	NCT02799602
	TRC253	Phase I/II	mCRPC and prostate adenocarcinoma	Active	NCT02987829
	TAS3681	Phase I	mCRPC (multinational study)	Recruiting	NCT02566772
	RAD140	Phase I	Hormone receptor positive malignant neoplasm of breast	Active	NCT03088527
	Proxalutamide (GT0918)	Phase II	mCRPC	Recruiting	NCT03899467
	Seviteronel (VT-464)	Phase II	CRPC	Completed	NCT02012920
	Enobosarm	Phase II	AR^+^ metastatic TNBC	Active	NCT02971761
GR	Relacorilant (CORT125134)	Phase I/II	Solid tumors in combination with nab-paclitaxel	Active	NCT02762981
	ORIC-101	Phase I	Advanced or metastatic solid tumors (in combination with nab-paclitaxel)	Recruiting	NCT03928314
PR	Onapristone	Phase I/II	Prostate cancer, Androgen-independent Prostate cancer (in combination with abiraterone)	Unknown	NCT02049190
	Telapristone acetate (CDB-4124)	Phase II	Stage 1A, 1B and 2 breast cancer	Active	NCT01800422
	Mifepristone	Not Applicable	Breast cancers with ratios of PRA/PRB higher than 1.5 and PR higher than 50%	Active	NCT02651844
RAR/RXR	IRX4204	Phase I	Previously treated advanced NSCLC (in combination with erlotinib)	Recruiting	NCT02991651
	9-cis-UAB-30	Phase I	Early-Stage Breast Carcinoma and Invasive Breast Carcinoma	Recruiting	NCT02876640
	Tamibarotene (SY-1425/Am80)	Phase II	Acute Myeloid Leukemia (as a monotherapy or in combination with azacytidine)	Active	NCT02807558
	Tretinoin	Phase I	Acute Myelogenous Leukemia (in combination with Tranylcypromine)	Active	NCT02273102
PPAR	Efatutazone (inolitazone, CS-7017, RS-5444) *(PPARγ agonist)*	Phase II	Previously treated myxoid liposarcoma that cannot be removed by surgery	Active	NCT02249949
	Pioglitazone Hydrochloride *(PPARγ agonist)*	Phase II	Head and Neck Cancer, Oral Leukoplakia	Completed	NCT00099021
	Iloprost *(PPARα/δ agonist)*	Phase II	Lung cancer, Precancerous Condition	Completed	NCT00084409
